# Letter to the Editor - ‘A decision tree model to help treatment decision-making for severe spontaneous intracerebral hemorrhage’

**DOI:** 10.1097/JS9.0000000000001488

**Published:** 2024-05-03

**Authors:** Hang-Tsung Liu, Ching-Hua Hsieh

**Affiliations:** Department of Trauma Surgery, Kaohsiung Chang Gung Memorial Hospital, and Chang Gung University, College of Medicine, Kaohsiung, Taiwan

*Dear Editor,*

We read with considerable interest the recent *International Journal of Surgery* article titled ‘A decision tree model to aid in treatment decision-making for severe spontaneous intracerebral hemorrhage’^1^. A decision tree model was developed in this study to forecast the postoperative prognosis of patients aged 18–75 who presented with severe spontaneous intracerebral hemorrhage. By considering factors such as renal dysfunction, ischemic cerebrocardiovascular disease history, hematoma volume, ischemic cerebrocardiovascular disease history, and Glasgow coma score (GCS) at admission, this model demonstrates a high level of predictive accuracy for 180-day poor outcomes. In the validation cohort, the AUCROC (area under the receiver operating characteristic curve) is 0.92. Moreover, this model has the potential to enhance the performance of junior clinicians in identifying patients who are at risk for adverse outcomes.

Although this decision tree model has a good predictive performance, using it goes against intuition and the typical method that a physician takes when dealing with patients. That is because this model separated the variables of GCS, ischemic cerebrocardiovascular disease history, and hematoma volume into different nodes in different layers of the model, making the model’s use difficult and not intuitive; thus, you must check the model repeatedly to ensure that the pathway continues. As a result, based on the author’s decision tree, we reformed it into a logistic algorithm (Figure 1) to provide a more reasonable and simple method for such patients in accordance with the downstream process. Notably, one variable used in the model is ‘ischemic cerebrovascular and cardiovascular diseases,’ which included transient ischemic attacks, ischemic stroke, coronary artery disease, and myocardial infarction. However, I felt that the use of ‘ischemic cerebrovascular or cardiovascular diseases’ for the node of variable would be more correct and less confusing.

**Figure 1:**
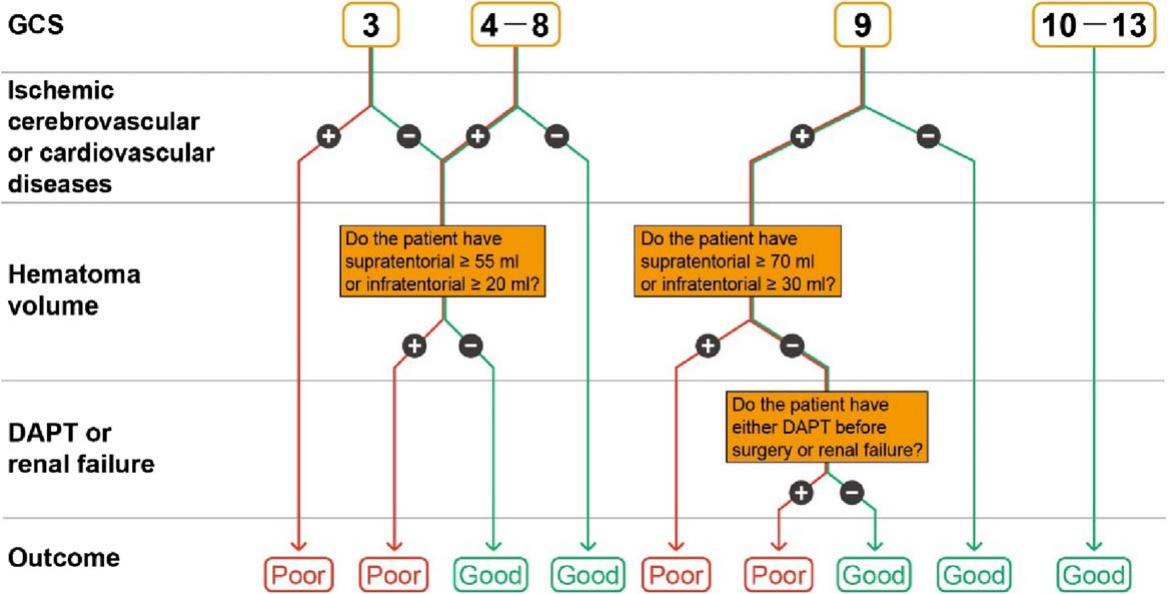
The reformed decision algorithm to determine the outcome of patients with severe spontaneous intracerebral hemorrhage. DAPT, dual antiplatelet therapy; GCS, Glasgow coma score.

## Ethical approval

Not applicable.

## Consent

Not applicable.

## Sources of funding

Not applicable.

## Author contribution

H.-T.L.: design of the updated decision algorithm; C.-H.H.: study concept and manuscript writing.

## Conflicts of interest disclosure

The authors declare no conflicts of interest.

## Research registration unique identifying number (UIN)

Not applicable.

## Guarantor

Both authors.

## Data availability statement

Not applicable.

## Provenance and peer review

Not commissioned, externally peer-reviewed.
